# Elevated oxygen extraction during heart transplantation is associated with increased morbidity and mortality: Implications for goal-directed perfusion

**DOI:** 10.1016/j.xjon.2025.101554

**Published:** 2025-12-08

**Authors:** Mark Petrovic, Awab Ahmad, Chen Chia Wang, Aaron M. Williams, John Trahanas, Swaroop Bommareddi, Tarek Absi, Eric Quintana, Kevin McGann, Stephen DeVries, Joshua Lowman, Aniket S. Rali, Hasan Siddiqi, Kaushik Amancherla, Stacy Tsai, Marshall Brinkley, Jonathan N. Menachem, Dawn Pedrotty, Lynn Punnoose, JoAnn Lindenfeld, Suzanne Sacks, Sandip Zalawadiya, Anthony Lepore, Matthew Warhoover, Matthew Bacchetta, Kelly Schlendorf, Ashish S. Shah, Brian Lima

**Affiliations:** a Department of Cardiac Surgery, Vanderbilt University School of Medicine, Nashville, Tenn; b Department of Cardiac Surgery, Vanderbilt University Medical Center, Nashville, Tenn; c Department of Biomedical Engineering, Vanderbilt University, Nashville, Tenn; d Department of Medicine, Vanderbilt University Medical Center, Nashville, Tenn

**Keywords:** heart transplantation, cardiopulmonary bypass, goal directed perfusion, oxygen delivery, oxygen extraction ratio

## Abstract

**Background:**

Goal-directed perfusion (GDP) during cardiopulmonary bypass (CPB) commonly targets indexed oxygen delivery (DO_2_i), yet fixed delivery thresholds may ignore patient-specific metabolic demand. The oxygen extraction ratio (O_2_ER) integrates delivery and consumption and may better reflect supply–demand balance during heart transplantation. We evaluated whether intra-CPB O_2_ER burden is associated with adverse outcomes after adult heart transplantation and whether O_2_ER provides incremental prognostic value beyond DO_2_i.

**Methods:**

We retrospectively analyzed adult heart transplantations performed at a single center between November 2021 and June 2025. Minute-level CPB data were extracted. O_2_ER was the primary exposure, and the primary outcome was a composite morbidity–mortality (M-M) endpoint (severe primary graft dysfunction [PGD], ventilation for >72 hours, intensive care unit length of stay >15 days, renal replacement therapy, or 90-day mortality). Generalized propensity score–weighted logistic regression modeled associations adjusting for prespecified donor/recipient/procedural covariates. Comparative models assessed O_2_ER versus DO_2_i. A post hoc analysis quantified pre- and post-reperfusion O_2_ER area under the receiver operating characteristic curve (AUC) to localize phase-specific risk.

**Results:**

Among 381 heart transplant recipients, 40 (10.5%) experienced M-M. O_2_ER trajectories separated between the M-M and non–M-M groups during the mid-procedure window (∼35-100 minutes). Each additional 10 minutes at O_2_ER > 0.20 was associated with higher odds of M-M (odds ratio [OR], 1.07; 95% confidence interval [CI], 1.00-1.15; *P* = .043) and 90-day mortality (OR, 1.13; 95% CI, 1.02-1.26; adjusted *P* = .02). Adding time at O_2_ER > 0.20 improved a DO_2_i < 280-only model (*P* = .04), whereas adding DO_2_i below-time to an O_2_ER-only model did not (*P* = .30). Phase-specific analysis showed that post-reperfusion O_2_ER AUC was independently associated with M-M (OR, 1.23; 95% CI, 1.08-1.40; *P* = .002) and severe PGD (OR, 1.22; 95% CI, 1.04-1.43; *P* = .01), while pre-reperfusion O_2_ER AUC was related to 90-day mortality (OR, 1.05; 95% CI, 1.004-1.10; *P* = .03).

**Conclusions:**

During heart transplantation, a higher O_2_ER burden on CPB is linearly associated with increased post-transplant morbidity and early mortality and contributes prognostic information beyond DO_2_i. These data support an O_2_ER-guided GDP strategy that minimizes time (or AUC) above O_2_ER thresholds, with heightened vigilance regarding reperfusion. Prospective validation is warranted.

## Graphical Abstract

**Figure:**
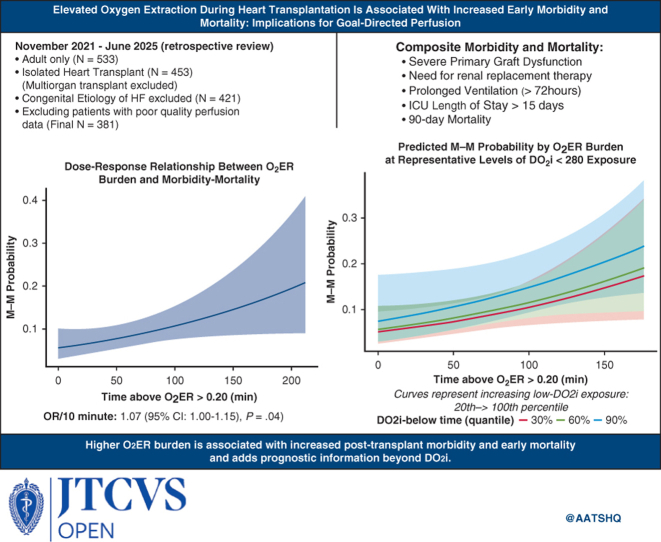

**Figure:**
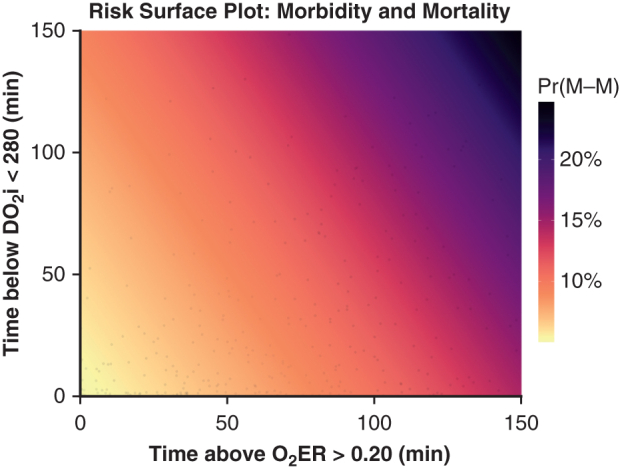
High O_2_ER (time at >0.2) is associated with post-transplant morbidity and mortality after adjusting for low DO_2_i (time at <280).

**Central Message**

Prolonged oxygen extraction ratio >0.20 during cardiopulmonary bypass shows a dose–response association with post-transplant morbidity and mortality, suggesting that perfusion optimization may improve heart transplant outcomes.

**Perspective**

This study demonstrates that prolonged oxygen extraction ratio >0.20 during cardiopulmonary bypass is associated with increased morbidity and early mortality after heart transplantation in a dose–response manner. These findings suggest that intraoperative perfusion optimization may represent a modifiable target for improving transplant outcomes.

Maintenance of adequate tissue oxygenation during cardiopulmonary bypass (CPB) is a critical determinant of outcomes in cardiac surgery. Historically, CPB has been guided by target cardiac index values^1^^,^^2^; however, evidence suggests that flow-based targets alone may be insufficient to ensure adequate tissue oxygen delivery.^3^^,^^4^ Recently, attention has shifted toward goal-directed perfusion (GDP), which incorporates multiple parameters during CPB, including pump flow, indexed oxygen delivery (DO_2_i), mean arterial pressure, mixed venous oxygen saturation (SvO_2_), and hematocrit to optimize systemic perfusion.^5^^,^^6^ Contemporary GDP practices focus on maintaining DO_2_i above a specific threshold of either 280 or 300 mL/min/m^2^ throughout CPB.^7^

GDP (ie, maintaining a targeted DO_2_i) has been associated with reduced renal dysfunction, decreased mild acute kidney injury,^4^^,^^5^^,^^7^, ^8^, ^9^, ^10^, ^11^, ^12^ and reduced morbidity.^13^, ^14^, ^15^ Two recent meta-analyses support these findings^16^^,^^17^; however, heart transplantation either represented a small fraction of cases or was excluded in these studies. Thus, despite the physiologic complexity of this procedure and the unique demands of graft reperfusion the application and optimization of GDP principles in adult heart transplantation remain poorly characterized.

Furthermore, a key limitation of DO_2_i-centric GDP is its focus on oxygen supply without explicitly accounting for metabolic demand. The O_2_ER integrates oxygen delivery and consumption, thereby indexing supply–demand balance.^18^ Outside the GDP literature, O_2_ER has been reported as a sensitive indicator of metabolic stress and impending organ injury, suggesting its potential utility as an operational target.^19^^,^^20^ As metabolic demand can vary widely individually, GDP in transplantation specifically and cardiac surgery in general should seek metabolic balance (ie, adjusting delivery in response to demand), implying that minimizing O_2_ER (rather than meeting a fixed DO_2_i threshold alone) may be a more physiologically grounded and patient-focused strategy during CPB, particularly regarding graft reperfusion.

This study evaluated whether CPB O_2_ER burden is associated with adverse outcomes after adult heart transplantation and whether O_2_ER provides incremental or superior prognostic information relative to DO_2_i alone, supporting a O_2_ER-guided perfusion strategy during CPB in heart transplant.

## Methods

### Study Design and Population

In this retrospective, single-center cohort study of adult heart transplantations performed at Vanderbilt University Medical Center between November 2021 and June 2025, exclusion criteria included multiorgan transplantation, adult congenital heart disease, and missing perfusion data. This study was deemed exempt by the Vanderbilt University Medical Center Institutional Review Board, with a waiver of informed consent granted (IRB #241909, approved January 1, 2025).

### Exposure

O_2_ER was defined as the ratio of the oxygen consumption index (VO_2_i) to the oxygen delivery index (DO_2_i), both captured continuously during CPB. As the primary dose–response exposure, we used time (minutes) with O_2_ER > 0.20, motivated by published physiologic ranges in which an O_2_ER ≈ 0.20 to 0.30 is considered normal.^18^ Time above a threshold is simple to track at the pump and reflects cumulative supply–demand imbalance.

### Outcomes

The primary outcome was a composite morbidity and mortality (M-M) outcome (see the Appendix E1 for rationale), comprising severe primary graft dysfunction (PGD) as defined by the 2014 International Society for Heart and Lung Transplantation Consensus Statement,^21^ post-transplant ventilation duration >72 hours, intensive care unit length of stay (LOS) >15 days, need for renal replacement therapy, and/or 90-day mortality. Secondary outcomes included severe PGD and 90-day mortality separately. Other outcomes, such as vasoactive-inotropic score at 24 hours post-transplantation and 72 hours post-transplantation, continuous renal replacement therapy, intensive care unit LOS, hospital LOS, and 1-year mortality, were reported as well.

### CPB Data Processing

Continuous minute-to-minute CPB data were extracted from the institutional perfusion data management system (Quantum Perfusion System; Spectrum Medical). Patients with more than 10 minutes of missing data were excluded. Missing values within the remaining CPB parameters were imputed using the k nearest-neighbor method. CPB time was segmented into pre-reperfusion and post-reperfusion phases, with reperfusion defined by removal of aortic cross-clamp in the recipient. The CPB duration was capped based on the median post-reperfusion CPB time to avoid confounding by post-reperfusion intraoperative graft dysfunction. The initial 5 minutes of CPB were excluded because of physiologic variability during CPB initiation.

### Statistical Analysis

This study adhered to the Strengthening the Reporting of Observational Studies in Epidemiology (STROBE) guidelines.

#### O_2_ER time-series analysis and baseline comparisons grouped by M–M outcome

Minute-level O_2_ER trajectories were modeled as the dependent variable and compared in patients with and those without the composite morbidity and mortality (M-M) outcome. Time was treated continuously with a natural cubic spline, and we included a time × M-M interaction to test for differing trajectories between groups. The mixed-effects model used a random intercept for patient (record id) to account for repeated measures within individuals. The spline basis dimension (k) was selected by minimizing the Akaike information criterion (AIC) and Bayesian information criterion (Appendix E1, Section E1). Baseline donor and recipient characteristics were then compared between groups. Categorical variables were summarized as counts (percentages) and compared using the χ^2^ test or Fisher exact test, as appropriate. Continuous variables were summarized as median with interquartile range [IQR] and compared using the Wilcoxon rank-sum test. Normality was assessed with the Shapiro-Wilk test and by inspection of Q-Q plots and histograms; continuous variables were non-normally distributed, supporting the use of nonparametric comparisons.

#### Dose–response association between time at O_2_ER >0.20 and outcomes

Weighted logistic regression was used to quantify the effect of time with O_2_ER >0.20 on the composite M-M outcome. Generalized propensity score weights for the continuous exposure were estimated conditional on donor age, female-to-male size mismatch, predicted heart mass ratio, prior sternotomy, left ventricular assist device (LVAD) explantation, pretransplantation extracorporeal membrane oxygenation (ECMO), procurement type (donation after brain death vs donation after circulatory death), storage strategy (ice, 10 °C static cold storage [SCS], machine perfusion, or 4 °C-8 °C SCS), and waitlist status; weights were trimmed at the 1st to 99th percentiles. Postweighting balance was confirmed by a reduction in the *R*^2^ from regressing exposure on covariates (*R*^2^ [T ∼ X]) toward 0, absolute weighted correlations (r) <0.10 for numerical covariates, and weighted omnibus tests with *P* ≥ .05 for categorical covariates; the effective sample size was reported as well (Section E3). The exposure–response was modeled both linearly and with a restricted cubic spline (3 degrees of freedom [df]) and compared using a likelihood ratio test (LRT) to test the ideal association. The same weighted approach was applied to severe PGD and 90-day mortality, with Holm correction for multiple secondary tests (2-sided α = 0.05). Furthermore, an L1 regularized regression model was built to assess the pretransplantation risk factors associated with prolonged time at O_2_ER >0.2 (Section E3).

#### Dichotomized O_2_ER burden (75th percentile) and outcome associations

Complementing the continuous exposure–response analysis, we dichotomized O_2_ER burden at the 75th percentile of time at O_2_ER >0.20 (high vs low). Propensity scores for the high-burden indicator were estimated using the same covariates as above, and inverse probability of treatment weighting was applied to balance baseline differences, targeting a postweighting standardized mean difference (SMD) <0.15 (Table E1). Associations with binary outcomes were estimated using weighted Firth-penalized logistic regression; differences in continuous outcomes were estimated using weighted quantile (median) regression. Across secondary endpoints, multiplicity was controlled using Holm adjustment (2-sided α = 0.05). One-year survival was compared between high- and low-burden groups using Kaplan-Meier curves and a log-rank test.

#### Joint O_2_ER–DO_2_i modeling and risk visualization

Logistic regression models were used to predict the composite M-M outcome from (1) time at O_2_ER >0.20 alone, (2) time at DO_2_i <280 alone, and (3) an additive model including both exposures. Multicollinearity was assessed with variance inflation factors (VIFs); a VIF <2 was prespecified as acceptable. An LRT compared the O_2_ER-only model with the additive model to evaluate the incremental value of adding DO_2_i. Discrimination was summarized by area under the receiver operating characteristic curve (AUC) with 95% confidence interval (CI), and AUCs were compared using the DeLong test. Overall fit and accuracy were described using AIC and the Brier score. To explore effect modification, an interaction term (O_2_ER × DO_2_i) was tested by LRT; because it was not significant, the additive specification was retained. For visualization, a model-based heat map (with overlaid probability contours) displayed the predicted risk of M-M across the joint grid of time at O_2_ER >0.20 and time at DO_2_i <280. In addition, conditional probability curves were plotted for O_2_ER exposure at fixed DO_2_i levels (30th, 60th, and 90th percentiles), with pointwise 95% confidence bands, to show how risk changes with O_2_ER at clinically representative DO_2_i burdens.

#### Phase-specific O_2_ER burden during CPB

A post hoc sensitivity analysis divided CPB into pre-reperfusion and post-reperfusion phases. Within each phase, the O_2_ER burden was summarized as area under the O_2_ER-time curve, AUC(O_2_ER), computed by trapezoidal integration over the phase window (units: O_2_ER × minutes) anchored to the documented clamp-off time. For each outcome (composite M-M, 90-day mortality, and severe PGD), 2 types of phase-specific logistic models were fit: univariable models with pre-reperfusion AUC(O_2_ER) alone and joint models including pre- and post-reperfusion AUC(O_2_ER) simultaneously. The incremental value of adding post-reperfusion AUC was evaluated with an LRT. Because these comparisons were post hoc, results are presented as exploratory. When multiple LRTs were considered across outcomes, Holm adjustment was applied to control the familywise error rate.

All statistical analyses and visualizations were conducted using R version 4.5.0 (R Foundation for Statistical Computing).

## Results

### O_2_ER Time-Series Analysis and Baseline Comparisons Grouped by M-M Outcome

**Figure 1:**
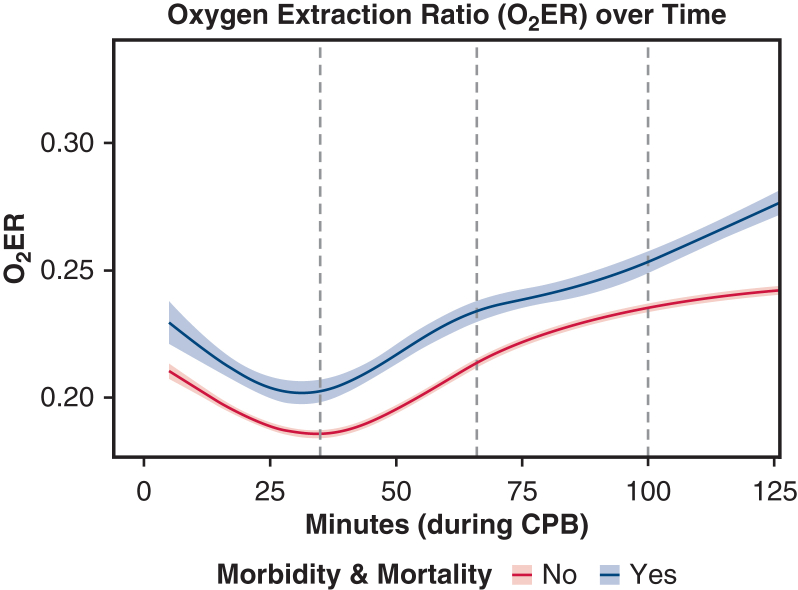
Intraoperative oxygen extraction ratio (O_2_ER) trajectories grouped by composite morbidity and mortality (M-M) outcome. A mixed-effects natural cubic spline model (knots placed at 35, 66, and 100 minutes) showed higher O_2_ER in the M-M group compared to the non–M-M group, especially during the mid-phase of cardiopulmonary bypass (∼35-100 minutes). Shaded bands indicated 95% confidence intervals. Change in O_2_ER in the M-M group compared to the non–M-M group at 0 to 35 minutes: −0.002 (*P* = .40); at 35 to 66 minutes: +0.04 (*P* < .001); at 66 to 100 minutes: +0.03 (*P* = .002); at >100 minutes: +0.006 (*P* = .74).

A total of 381 adult heart transplant recipients were included, of whom 40 (10.5%) experienced an M-M outcome and 341 (89.5%) did not. An average of 5.3% of the CPB data per patient was missing, which was imputed. Using a natural cubic spline (internal knots at 35, 66, and 100 minutes), O_2_ER trajectories differed between patients with and without M-M specifically in the mid-procedure window (∼35 to 100 minutes), as indicated by significant interaction terms for the second and third spline bases (*P* < .001 and *P* = .0015, respectively). Early (≤35 minutes) and late (≥100 minutes) phases did not show significant between-group differences. The main effect of group at the spline reference point was not significant (*P* = .223), indicating that the difference is time-dependent rather than constant (Figure 1). Baseline characteristics stratified by M-M outcome are summarized in Table 1. The M-M group had a significantly higher average O_2_ER (M-M: 0.25 [IQR, 0.20-0.27] vs no M-M: 0.22 [IQR, 0.17-0.26]; *P* = .02), AUC (29.38 [IQR, 22.43-36.88] vs 24.96 [IQR, 18.69-32.38]; *P* = .02), time at O_2_ER >0.20 (86.1 [IQR, 55.21-114.32] minutes vs 64.4 [IQR, 30.5-102] minutes; *P* = .006), and O_2_ER area >0.2 (6.37 [IQR, 3.07-9.34] vs 3.19 [IQR, 0.87-7.31]; *P* = .005). Other differences included higher rates of pretransplantation LVAD, ECMO, and prior sternotomy and prolonged allograft ischemic time (*P* < .05 for all).

**Table 1:** Baseline donor and recipient characteristics

Characteristic	Total (N = 381)	M-M absent (N = 341)	M-M present (N = 40)	*P* value
Recipient characteristics				
Age, y, median (IQR)	58.4 (47.4-64.9)	59.10 (47.11-64.86)	56.01 (47.57-64.31)	.63
Female sex, n (%)	93 (24.4)	82 (24.0)	11 (27.5)	.70
BMI, median (IQR)	29.67 (25.96-33.68)	29.61 (25.88-33.66)	30.32 (27.32-33.85)	.18
Hypertension, n (%)	239 (62.7)	214 (62.8)	25 (62.5)	>.99
Diabetes mellitus, n (%)	149 (39.1)	133 (39.0)	16 (40.0)	>.99
Ischemic cardiomyopathy, n (%)	107 (28.1)	96 (28.2)	11 (27.5)	>.99
Waitlist status, n (%)				.06
Status 1	29 (7.6)	23 (6.7)	6 (15)	
Status 2	86 (22.6)	82 (24.0)	4 (10.0)	
Status 3	96 (25.2)	87 (25.5)	9 (22.5)	
Status 4	99 (26.0)	84 (24.6)	15 (37.5)	
Status 6	71 (18.6)	65 (19.1)	6 (15.0)	
LVAD pretransplantation, n (%)	113 (29.7)	95 (27.9)	18 (45.0)	**.03**
ECMO pretransplantation, n (%)	13 (3.4)	9 (2.6)	4 (10)	**.04**
Prior sternotomy, n (%)	196 (51.4)	167 (49.0)	29 (72.5)	**.007**
Prehospitalization status, n (%)	166 (43.6)	151 (44.3)	15 (37.5)	.51
Pretransplant creatinine, median (IQR)	1.20 (0.94-1.47)	1.20 (0.94-1.45)	1.27 (1.04-1.52)	.52
Female-to-male mismatch, n (%)	51 (13.4)	49 (14.4)	2 (5.0)	.14
Recovery parameters				
Procurement, n (%)				.41
DCD	172 (45.1)	190 (55.7)	19 (47.5)	
DBD	209 (54.9)	151 (44.3)	21 (52.5)	
Total ischemic time, min, median (IQR)	234 (201-271)	232 (200-267)	261.5 (214.5-308)	**.02**
Allograft recovery, n (%)				.051
Ice	137 (36.0)	125 (36.7)	12 (30.0)	
10 °C cooler	195 (51.2)	177 (51.9)	18 (45.0)	
NMP	23 (6.0)	20 (5.9)	3 (7.5)	
HMP	24 (6.3)	18 (5.3)	5 (4.2)	
4 °C-8 °C SCS	2 (0.5)	1 (0.3)	1 (2.5)	
Donor characteristics				
Age, y, median (IQR)	33 (25-42)	33 (25-42)	33 (25-42.4)	.97
Female sex, n (%)	116 (30.4)	106 (31.1)	10 (25.0)	.58
BMI, median (IQR)	26.64 (22.72-32.01)	26.57 (22.58-31.80)	28.56 (23.83-36.12)	.12
Hypertension, n (%)	81 (21.3)	74 (21.7)	7 (17.5)	.68
PHM ratio, median (IQR)	0.93 (0.83-1.08)	0.92 (0.83-1.06)	0.96 (0.83-1.12)	.54
Donor LVEF, %, median (IQR)	60 (55-65)	60 (55-65)	60 (60-67)	.21
Donor distance, nautical mi, median (IQR)	317.4 (150.2-456.2)	317.4 (153.7-456.2)	347.8 (139.4-528.8)	.58
Cause of death, n (%)				.31
CVA/ICH	55 (14.4)	7 (9.6)	5 (12.5)	
Blunt trauma	83 (21.8)	27 (37.0)	13 (32.5)	
Hypoxia/anoxia	141 (37.0)	21 (28.8)	15 (37.5)	
Other	102 (26.7)	95 (27.9)	7 (17.5)	
Cardiac arrest/CPR attempt, n (%)	203 (53.3)	183 (53.7)	20 (50)	.74
O_2_ER dose–response metrics during transplant				
Median (IQR)	0.22 (0.17-0.27)	0.22 (0.17-0.26)	0.25 (0.20-0.27)	**.02**
AUC, median (IQR)	25.36 (18.89-32.98)	24.96 (18.69-32.38)	29.38 (22.43-36.88)	**.02**
Time above, median (IQR)				
0.20	67.9 (33.03-105)	64.4 (30.5-102)	86.1 (55.21-114.32)	**.006**
0.25	32.2 (5.16-64.46)	29.96 (3.95-64.36)	47.51 (23.30-71.52)	**.01**
0.30	4.19 (0.00-26.62)	3.33 (0.00-23.38)	15.96 (2.88-33.94)	**.01**
Area above, median (IQR)				
0.20	3.39 (1.04-7.78)	3.19 (0.87-7.31)	6.37 (3.07-9.34)	**.005**
0.25	1.04 (0.08-3.06)	0.91 (0.06-2.84)	2.21 (0.70-4.25)	**.003**
0.30	0.08 (0.00-0.71)	0.05 (0.00-0.66)	0.33 (0.06-1.64)	**.007**
CPB length, min, median (IQR)	144 (106.25-175)	141 (105-170)	175 (123.5-223)	**.001**
CPB length capped, min, median (IQR)	124.6 (99.8-149.5)	124.33 (99.5-145.33)	131.3 (105.3-167.5)	.21

Bold type indicates significance.

*M-M*, Morbidity and mortality; *BMI*, body mass index; *ECMO*, extracorporeal membrane oxygenation; *DCD*, donation after circulatory death; *DBD*, donation after brain death; *NMP*, normothermic machine perfusion; *HMP*, hypothermic machine perfusion; *SCS*, static cold storage; *PHM*, predicted heart mass; *LVEF*, left ventricular ejection fraction; *CVA*, cerebrovascular accident; *ICH*, intracranial hemorrhage; *CPR*, cardiopulmonary resuscitation; *AUC*, area under the curve; *CPB*, cardiopulmonary bypass.

### Dose–Response Association Between Time at O_2_ER >0.20 and Outcomes

**Figure 2:**
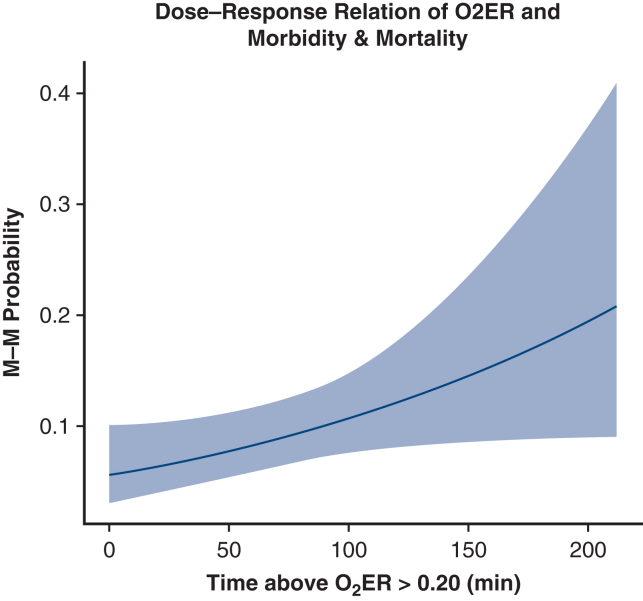
Dose–response of oxygen extraction ratio (O_2_ER) burden and composite morbidity and mortality (M-M). Predicted probability of post-transplant M-M as a function of time at O_2_ER >0.2 (minutes). The curve shows a linear dose–response relationship of increasing probability of M-M with increasing time at O_2_ER >0.2 during cardiopulmonary bypass.

In weighted logistic regression adjusting for confounders, each additional 10 minutes with O_2_ER > 0.20 was associated with a 7% higher odds of experiencing the composite M-M outcome (odds ratio [OR], 1.07, 95% CI, 1.00-1.15; *P* = .043) (Figure 2). Modeling the exposure with natural splines (df = 3) did not improve fit (ΔDeviance = 1.68; df = 2; *P* = .43; AIC linear 242.4 vs spline 244.5), supporting a linear dose–response. Similarly, a 10-minute increase in time at O_2_ER >0.20 was associated with higher 90-day mortality; the odds increased by 13% per 10 minutes (OR, 1.13; 95% CI, 1.02-1.26; adjusted *P* = .02), but there was no significant association with severe PGD (OR, 1.00; 95% CI, 0.92-1.09; adjusted *P* = .96). The L1 regularized regression model found that a history of diabetes, prior sternotomy, and pretransplantation LVAD and ECMO were associated with prolonged time at O_2_ER > 0.20, while allograft storage using 10 °C SCS and hypothermic oxygenated perfusion had a negative association (Section E4).

### Dichotomized O_2_ER Burden (75th Percentile) and Outcome Associations

**Figure 3:**
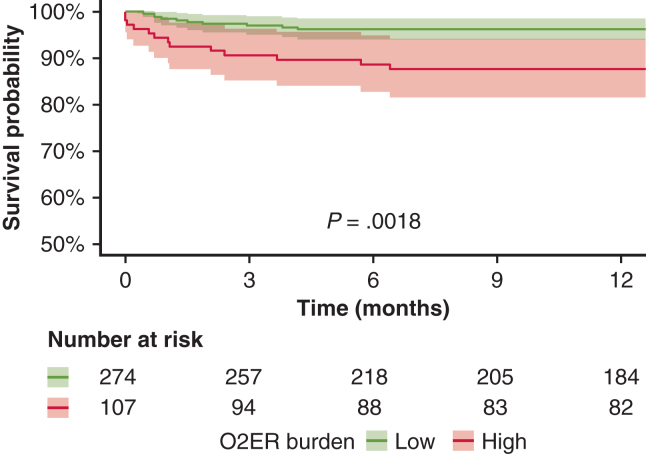
Kaplan Meier survival curves (with 95% confidence intervals [CIs]) showing 1-year survival differences between the high oxygen extraction ratio (O_2_ER) burden group (>75th percentile of time at O_2_ER >0.2) and the low O_2_ER burden group. The high O_2_ER group had significantly worse 1-year survival (87.7%; 95% CI, 81.6%-94.2%) compared to the low O_2_ER group (96.3%; 95% CI, 94.0%-98.6%) (log-rank *P* = .0018).

Dividing the population by 75th percentile of time at O_2_ER >0.20 (ie, high vs low O_2_ER burden) revealed that high O_2_ER burden was independently associated with M-M (OR, 1.69; 95% CI, 1.09-2.67; *P* = .02). It also was associated with severe PGD (OR, 1.96; 95% CI, 1.12-3.56; *P* = .04), 90-day mortality (OR, 2.23; 95% CI, 1.16-5.21; *P* = .003), and post-CPB severe right ventricular dysfunction (OR, 4.48; 95% CI, 1.90-12.36; *P* = .001) (Tables 2 and E2). The high O_2_ER burden group also had significantly worse 1-year survival (87.7%; 95% CI, 81.6%-94.2%) compared to the low O_2_ER burden group (96.3%; 95% CI, 94.0%-98.6%; *P* = .002) (Figure 3).

**Table 2:** Adjusted association of high O_2_ER burden compared to low O_2_ER burden (reference)

Variable	OR (95% CI)/median change (95% CI)	*P* value	Holms-adjusted *P* value
M-M	1.69 (1.09-2.67)	**.02**	---
Severe RV dysfunction	4.48 (1.90-12.36)	<.001	**.001**
Severe PGD	1.96 (1.12-3.56)	.019	**.038**
Mortality at 90 d	2.23 (1.16-5.21)	.001	**.003**
VIS at 24 h	2.00 (0.1-3.9)	.038	.23
VIS at 72 h	2.15 (−0.93 to 5.22)	.16	.48
Cardiac index at 24 h	0.10 (−0.24 to 0.44)	.56	.67
Cardiac index at 72 h	−0.07 (−0.29 to 0.15)	.49	.67
LVEF on POD 7 < 55%	2.05 (1.36-3.12)	<.001	**.001**
RRT	1.10 (0.78-1.56)	.55	.55
ICU LOS, d	0 (−1.85 to 1.85)	>.99	>.99
Hospital LOS, d	−1.0 (−3.49 to 1.49)	.43	.67

High oxygen extraction ratio (O_2_ER) burden is defined as >75th percentile of time at O_2_ER > 0.2. Bold type indicates significance.

*OR*, Odds ratio; *CI*, confidence interval; *RV*, right ventricular; *PGD*, primary graft dysfunction; *VIS*, vasoactive inotropic score; *POD*, postoperative day; *RRT*, renal replacement therapy; *ICU*, intensive care unit; *LOS*, length of stay.

### Joint O_2_ER–DO_2_i Modeling and Risk Visualization

**Figure 4:**
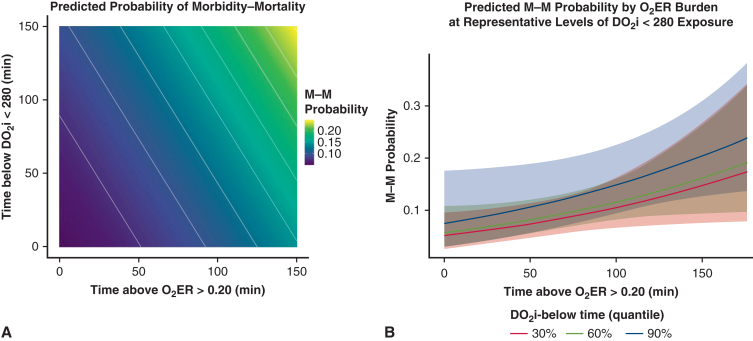
A, Heatmap of predicted composite morbidity and mortality (M-M) probability across the joint grid of increasing oxygen extraction ratio (O_2_ER) burden (time at O_2_ER >0.2 [*x*-axis]) and indexed oxygen delivery (DO_2_i) burden (time at DO_2_i <280 [*y*-axis]) from an additive logistic model (O_2_ER + DO_2_i). The highest-risk clusters at high O_2_ER (right side of the heatmap; *P* = .04), and high DO_2_i burden in the absence of O_2_ER burden does not increase the risk of M-M (left side of the heatmap; *P* = .29). B, Conditional risk curves for increasing O_2_ER >0.20 time during CPB at fixed DO_2_i below-time levels corresponding to the 30th, 60th, and 90th cohort percentiles. Shaded bands represent 95% confidence intervals. These curves indicate that M-M risk increases (*P* = .04) irrespective of DO_2_i burden (*P* = .29).

Using DO_2_i <280 minutes as the delivery burden, VIFs were low (both 1.24), indicating negligible collinearity. Discrimination was modest and similar across models. Adding time at O_2_ER >0.20 to a DO_2_i-only model improved fit (LRT *P* = .04), whereas adding time at DO_2_i <280 to an O_2_ER-only model did not (LRT *P* = .30). The O_2_ER × DO_2_i interaction was not significant (*P* = .89). In the additive model, each 10-minute increase in O_2_ER >0.20 corresponded to ∼9% higher odds of M-M (OR ≈ 1.09; *P* = .09), while DO_2_i <280 showed no clear association (OR ≈ 1.06; *P* = .53) suggesting that O_2_ER exposure carries the dominant signal, with limited incremental value from DO_2_i below time once O_2_ER is accounted for (Figure 4).

### Phase-Specific O_2_ER Burden During CPB

A post hoc sensitivity analysis was conducted dividing CPB into pre-reperfusion and post-reperfusion phases, and the O_2_ER burden was estimated using AUC. Pre-reperfusion AUC(O_2_ER) alone showed a borderline association with M-M (OR, 1.03; 95% CI, 0.95-1.86; *P* = .093). When post-reperfusion AUC(O_2_ER) was added, it was independently associated with higher odds of M-M (OR, 1.23; 95% CI, 1.08-1.40; *P* = .002), while pre-reperfusion was not (OR, 1.00; 95% CI, 0.95-1.04; *P* = .82). Model fit improved; LRT indicated significant incremental value for adding post-clamp (adjusted *P* = .003). For 90-day mortality, pre-reperfusion AUC(O_2_ER) alone had a strong association (OR, 1.05; 95% CI, 1.004-1.10; *P* = .03). In the joint model, adding post-reperfusion did not improve fit (LRT adjusted *P* = .361); however, for severe PGD, pre-reperfusion AUC(O_2_ER) alone was not associated (OR, 1.22; 95% CI, 1.04-1.43; *P* = .63), while in the joint model, post-reperfusion AUC(O_2_ER) was associated with significantly higher odds of severe PGD (OR, 1.22; 95% CI, 1.04-1.43; *P* = .01) but pre-reperfusion was not (OR, 0.98; 95% CI, 0.92-1.03; *P* = .39), and model fit improved (LRT for adding post-reperfusion, adjusted *P* = .02).

## Discussion

Heart transplantation presents unique physiologic challenges due to unique metabolic demands in the transplant population coupled with the complex hemodynamic changes following reperfusion of the implanted allograft. In this context, this study identifies the O_2_ER, a real-time index of supply–demand balance during CPB, as a pragmatic predictor of early outcomes after heart transplantation. The principal findings of the study can be summarized as follows. First, O_2_ER trajectories diverged between patients with and without the composite M-M outcome specifically during the mid-procedure window (∼35 to 100 minutes into CPB), indicating a time-dependent rather than constant separation. Second, each additional 10 minutes with O_2_ER >0.2 was associated with 7% higher odds of M-M and 13% higher odds of 90-day mortality. Third, O_2_ER carried the dominant prognostic signal; adding time at O_2_ER >0.20 improved a DO_2_i <280-only model (LRT *P* = .04), whereas adding DO_2_i <280 to an O_2_ER-only model did not (LRT *P* = .30). Finally, phase-specific sensitivity analyses further localized risk to reperfusion; post-reperfusion AUC(O_2_ER) was independently associated with both M-M and severe PGD, whereas pre-reperfusion AUC(O_2_ER) contributed little once post-reperfusion burden was included. Pre-reperfusion AUC(O_2_ER) alone was related to 90-day mortality. These findings support O_2_ER as a pragmatic, metabolically grounded target for transplant perfusion. Unlike DO_2_i, an index of supply, O_2_ER integrates supply and demand.

Traditional CPB management relies on perfusionist experience, SvO_2_, and cardiac index targets (eg, 1.8-2.4 L/min/m^2^),^1^ but these flow-based metrics might not adequately reflect tissue oxygen delivery and metabolic demand.^3^ GDP has emerged to optimize tissue oxygenation during CPB, with growing evidence supporting improved outcomes in elective cardiac surgery.^4^^,^^7^, ^8^, ^9^, ^10^, ^11^, ^12^ The GIFT trial demonstrated that maintaining a DO_2_i threshold of approximately 280 mL O_2_/min/m^2^ significantly reduced AKIN stage 1 acute kidney injury,^7^ while another study found that deviations from a GDP protocol targeting the same DO_2_i threshold were associated with prolonged mechanical ventilation and increased postoperative LOS.^15^ Magruder and colleagues^22^ reported similar results in heart transplantation, showing a link between inadequate DO_2_i and severe PGD, but they did not adjust for the intraoperative CPB time/phase linked to intraoperative PGD.

Although DO_2_i is a more widely studied metric, we chose O_2_ER as our primary parameter for monitoring oxygen debt because it accounts for oxygen demand.^20^ This is important, as metabolic demand can vary based on preoperative factors and the condition of the donor allograft. In transplant recipients, this means DO_2_i should be tailored to VO_2_i, and using a universal DO_2_i threshold without considering VO_2_i may be misleading. Furthermore, more sophisticated approaches targeting O_2_ER <0.25 have shown superior correlation with reduced hyperlactatemia, which is independently associated with morbidity following cardiac surgery, compared to traditional SvO_2_-guided protocols.^14^ Our results extend this literature by demonstrating, in transplantation, that the mismatch metric O_2_ER better explains risk than supply alone, and that the clinically pivotal period is reperfusion. Taken together, these data argue for GDP that targets metabolic balance (minimizing time/AUC above O_2_ER thresholds) rather than fixed delivery cutoffs alone.

The temporal pattern that we observed is physiologically coherent. The mid-procedure divergence in O_2_ER trajectories between the M-M and non–M-M cohorts aligns with periods of rewarming, flow redistribution, and changing oxygen consumption.^23^ Patients who fail to restore their supply–demand balance during this interval accrue “oxygen debt,” reflected by higher O_2_ER and worse outcomes. The dominance of O_2_ER over DO_2_i below time suggests that absolute delivery thresholds may be insufficient in isolation when metabolic demand rises. The selective association of post-reperfusion O_2_ER with M-M and PGD is biologically plausible; reperfusion magnifies oxygen demand and microcirculatory heterogeneity, amplifying any mismatch, coupled with potential free radical injury owing to restored allograft perfusion after a period of ischemia.^23^^,^^24^ Conversely, the link between pre-reperfusion O_2_ER and mortality suggests that recipient-side physiology before graft reperfusion may prime early hazards. In our cohort, patients with markers of higher illness burden (eg, LVAD, ECMO, ischemic cardiomyopathy, prior sternotomy, diabetes)^25^, ^26^, ^27^, ^28^ were overrepresented among those accruing a high intraoperative O_2_ER burden, which in turn independently tracked with severe PGD, early mortality, post-CPB right ventricular dysfunction, and worse 1-year survival (Section E4).

Although exploratory, this chain from preoperative fragility to intraoperative metabolic stress to early adverse outcomes offers a coherent narrative for risk localization and further supports that metabolic demand varies across patients and that one specific threshold might not be sufficient for all. Interestingly, 10 °C SCS and hypothermic oxygenated perfusion strategies were protective against O_2_ER burden, which may indicate better preservation of metabolic reserve and reduced ischemia-reperfusion damage (Section E4).

Our findings may inform GDP strategies across cardiac procedures and extracorporeal support. O_2_ER's actionability and consistent incremental value support its use as a real-time control variable rather than a stand-alone predictor. A practical GDP strategy in transplantation could (1) continuously display O_2_ER alongside DO_2_i, (2) adopt a “time-in-target” metric (eg, minimize minutes with O_2_ER >0.20), and (3) intervene with levers that reduce O_2_ER: raise pump flow, optimize hematocrit, ensure adequate mean arterial pressure without excessive vasoconstriction, adjust temperature/rewarming rate, deepen anesthesia/neuromuscular blockade to lower demand, and correct hypoxemia. The phase-specific signal suggests the need for heightened vigilance and predefined response bundles around reperfusion. These principles also may extend to other forms of mechanical circulatory support, including venoarterial ECMO.^29^

Future research directions should focus on several key areas to advance GDP implementation in heart transplantation. Prospective studies incorporating real-time intraoperative variables, including blood product utilization, intraoperative vasoactive support requirements, biomarkers, and continuous assessment of intraoperative and postoperative hemodynamics as more sensitive indicators of graft dysfunction,^30^ will provide a more nuanced understanding of perfusion optimization. Additionally, given the heterogeneity of the transplant population, investigation of donor-specific and recipient-specific GDP thresholds is warranted. This includes dedicated analyses for recipients with preexisting end-organ dysfunction and those undergoing multiorgan transplant, with a complex mechanical circulatory support history, or with congenital heart disease. Finally, multicenter randomized controlled trials, while challenging in this population, remain the gold standard for validating these findings and establishing GDP as a standard of care in heart transplantation.

Our study has several limitations, including the retrospective single-center design with residual confounding despite weighting, modest event counts, and potential measurement error in machine-derived VO_2_i and DO_2_i (hence O_2_ER). It is also important to note that our DO_2_i and VO_2_i values were console-derived. After cross-clamp removal, total flow equals pump plus native cardiac output, but the console uses pump flow only. As a result, DO_2_i and VO_2_i are underestimated during weaning. Our primary exposure, O_2_ER = VO_2_i/DO_2_i, is less affected because the common flow term cancels; O_2_ER reflects mainly arterial/venous saturation and hemoglobin. The remaining limitation is venous sampling; during weaning, the venous line might not capture a perfectly mixed whole-body sample, adding random noise, but such noise would be expected to dilute (attenuate) true associations rather than create spurious ones.

Despite these limitations, our analysis leverages continuous CPB waveforms in 381 transplants, time-resolved mixed-effects splines to localize risk to reperfusion, and a metabolically grounded exposure (O_2_ER) evaluated alongside DO_2_i with convergent tests (weighted models, LRTs, AUC/Brier/AIC, visual heat maps) and correction for multiple comparisons. These exploratory findings warrant external validation and prospective testing of O_2_ER-guided GDP.

In conclusion, high O_2_ER burden (representing supply–demand mismatch) during heart transplantation is strongly associated with post-transplant morbidity and mortality, including severe PGD and early mortality.

## Conflict of Interest Statement

The authors reported no conflicts of interest.

The *Journal* policy requires editors and reviewers to disclose conflicts of interest and to decline handling or reviewing manuscripts for which they may have a conflict of interest. The editors and reviewers of this article have no conflicts of interest.

## Figures and Tables

**Table E1:** Inverse probability of treatment weighting based on propensity scores estimated from gradient boosted modeling

Variable	SMD before balancing, %	SMD after balancing, %
Allograft ischemic time	24.1	8.8
Ischemic cardiomyopathy	10.3	1.6
Prior sternotomy	23.3	0.03
Ice	24	1.5
10 °C SCS	21.7	2.1
NMP	2.3	0.07
HMP	0.7	0.8
Pretransplant ECMO	0.5	0.4
Pretransplant LVAD	21.1	0.2
Diabetes	5.3	1.6

A standardized mean difference (SMD) of <15% was considered balanced. *SCS*, Static cold storage; *NMP*, normothermic machine perfusion; *HMP*, hypothermic machine perfusion; *ECMO*, extracorporeal membrane oxygenation; *LVAD*, left ventricular assist device.

**Table E2:** Post-transplant outcomes comparison in unadjusted population

Perioperative details	Total (N = 381)	Time at O_2_ER ≤ 0.2 (≤105 min) (N = 274)	Time at O_2_ER >0.2 (>105 min) (N = 107)	*P* value
Morbidity and mortality, n (%)	40 (10.5)	23 (8.4)	17 (15.9)	**.04**
Post-CPB LVEF <55%, n (%)	47 (12.3)	32 (11.7)	15 (14.0)	.14
Post-CPB severe RV depression, n (%)	11 (2.9)	4 (1.5)	7 (6.5)	**.01**
Severe PGD, n (%)	25 (6.6)	14 (5.1)	11 (10.3)	.10
VIS at 24 h, median (IQR)	11.32 (8.06-15.28)	10.53 (8.00-14.74)	13.00 (9.56-15.67)	**.01**
VIS at 72 h, median (IQR)	6.54 (4.00-10.94)	5.86 (4.00-9.97)	7.04 (4.00-11.86)	.11
Cardiac index at 24 h, median (IQR)	3.10 (2.69-3.66)	3.10 (2.69-3.63)	3.17 (2.64-3.68)	.84
Cardiac index at 72 h, median (IQR)	3.20 (2.70-3.60)	3.19 (2.70-3.60)	3.20 (2.71-3.60)	.85
LVEF at POD 7 < 55%, n (%)	52 (13.6)	28 (10.2)	24 (22.4)	**.002**
RRT, n (%)	82 (21.5)	56 (20.4)	26 (24.3)	.41
ICU LOS, d, median (IQR)	8 (5-12)	7 (5-12)	8 (6-12)	.32
Hospital LOS, d, median (IQR)	18 (13-26)	18 (13-25)	18 (14.5-27.5)	.43
30-d mortality, n (%)	10 (2.6)	4 (1.5)	6 (5.6)	**.03**
90-d mortality, n (%)	18 (4.7)	8 (2.9)	10 (9.3)	**.01**
1-y mortality, n (%)	23 (6.0)	10 (3.6)	13 (12.1)	**.003**

Bold values indicate statistical significance as *P* < .05.

*O*_*2*_*ER*, Oxygen extraction ratio; *CPB*, cardiopulmonary bypass; *LVEF*, left ventricular ejection fraction; *RV*, right ventricular; *PGD*, primary graft dysfunction; *VIS*, vasoactive-inotropic score; *IQR*, interquartile range; *POD*, postoperative day; *RRT*, renal replacement therapy; *ICU*, intensive care unit; *LOS*, length of stay.

**Table:** 

Variable	Score
Severe PGD	3
Need for RRT	1.5
Post-transplant ventilation time >72 h	2
ICU LOS >15 d	1.5
Mortality at 90 d	4
Total score	12

**Figure:**
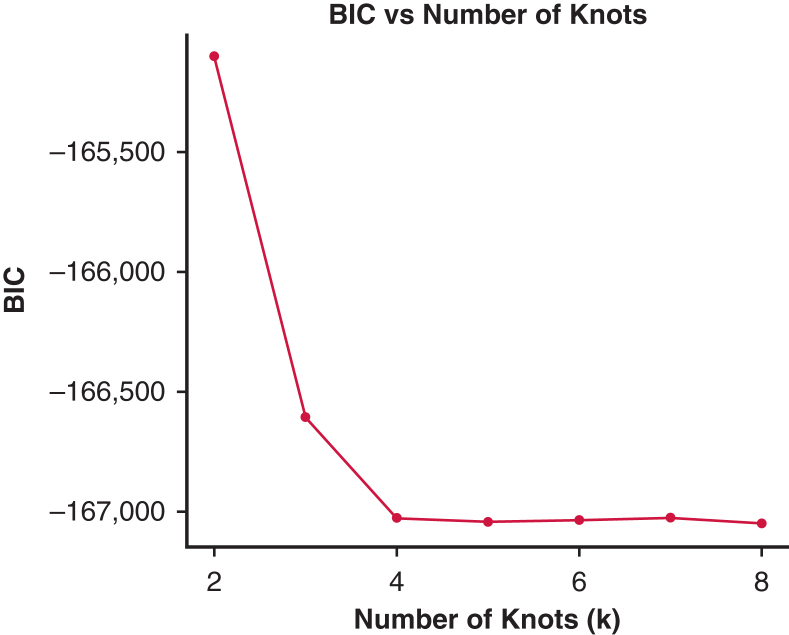


**Figure:**
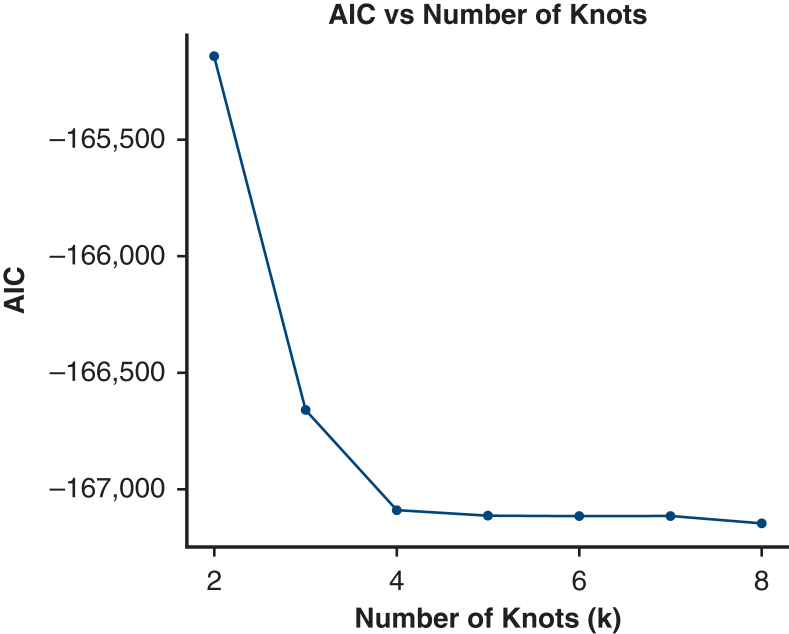


**Table:** 

Variable	Metric	Value
Storage strategy	ANOVA *P* value	.86
Donor age	Weighted correlation	−0.06
Female-to-male mismatch	ANOVA *P* value	.88
PHM ratio	Weighted correlation	−0.01
Procurement type (DBD vs DCD)	ANOVA *P* value	.83
Pretransplant LVAD	ANOVA *P* value	.73
Pretransplant ECMO	ANOVA *P* value	.07
Waitlist status	ANOVA *P* value	.38
Allograft ischemic time	Weighted correlation	0.02
Prior sternotomy	ANOVA *P* value	.08

*PHM*, predicted heart mass; *DBD*, donation after brain death; *DCD*, donation after circulatory death; *LVAD*, left ventricular assist device; *ECMO*, extracorporeal membrane oxygenation.

**Figure:**
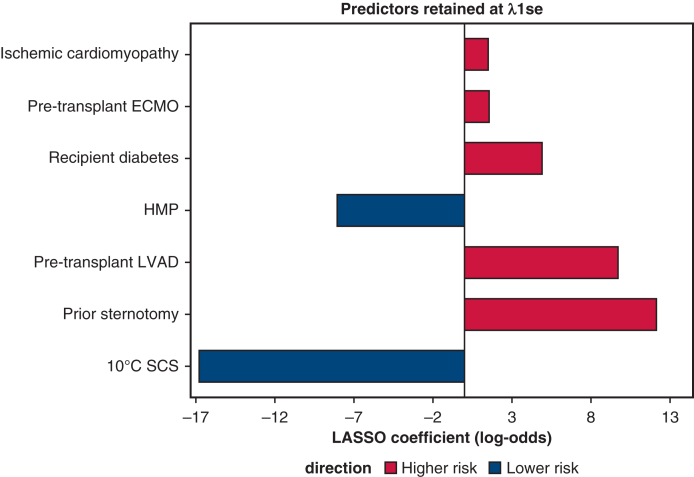
*HMP*, hypothermic oxygenated machine perfusion; *SCS*, static cold storage.

## Appendix E1

### Section E1: Composite Morbidity and Mortality

A score was developed to quantify the severity of postoperative adverse events following heart transplantation. Because not all complications carry the same prognostic weight, a weighted scoring system was constructed based on association with 90-day mortality, which served as the terminal event in this severity scale.

To determine the relative contribution of each event, univariable logistic regression models were used to assess their association with 90-day mortality. The postoperative events evaluated included.

• Severe primary graft dysfunction (PGD)
• Left ventricular ejection fraction (LVEF) < 55% on postoperative day 7
• Vasoactive-inotropic score (VIS) > 10 at 24 hours post-transplant
• Requirement for renal replacement therapy (RRT)
• Use of an intra-aortic balloon pump (IABP) postoperatively
• Post-transplant ventilation time >72 hours
• Intensive care unit (ICU) length of stay (LOS) >15 days

Among these, severe PGD, prolonged ICU LOS (>15 days), need for RRT, and post-transplant ventilation time >72 hours were significantly associated with 90-day mortality. The severity score assigned to each of these factors was determined based on their regression model coefficients.

To simplify the scoring system (ranging from 0 to 12), 90-day mortality, as the most critical outcome, was assigned the highest score of 4. This approach ensures that the scale meaningfully reflects the impact of adverse events on patient survival.

After assigning the score to each patient, composite morbidity and mortality outcome was created by grouping patients who had a score ≥4 to ensure that a high burden of post-transplant events is captured.

### Section E2: Linear Mixed-Effects Model With Cubic Splines

Formula: lmer (O_2_ER ∼ ns (minute, 4)∗M-M + (1 | record_id)

Optimal number of knots.

There was limited improvement beyond *k* = 4, so 4 was selected as the optimal degree of freedom for cubic splines.

### Section E3: Balance Diagnostics for Generalized Propensity Score for Weighted Logistic Regression

1. Balance for categorical variables was assessed with an analysis of variance (ANOVA) *P* > .05, and the correlation coefficient for continuous variables was <0.10.

2. Adjusted *R*^2^ reduced from 0.168 (unweighted) to 0.02 (weighted).

3. Effect sample size 300/381 (78.7%).

### Section E4. Independent Risk Factors for Prolonged Time at O_2_ER >0.2

We identified risk factors using L1-penalized logistic regression (LASSO) with 5-fold cross-validation, selecting the penalty via the 1-SE criterion; predictors were standardized, and variables with nonzero coefficients were retained.

Impact of high O_2_ER burden on post-transplant outcomes:

High O_2_ER burden defined as >3rd quartile of time at O_2_ER >0.2 (ie > 105 minutes).

### Section E5. Time Dose–Response Metrics Calculations

We analyzed continuous time-series for O_2_ER (primary) and DO_2_i (supporting). For each patient, we computed time above prespecified O_2_ER thresholds (0.20, 0.22, 0.25, and 0.30) and time below a DO_2_i threshold of 280 by scanning consecutive observations (t_i_, x_i_) and (t_i+1_, x_i+1_). If both values lay on the same side of the threshold τ, we added the full interval, Δt = t_{i+1}_ - t_i_. If the segment crossed τ, we linearly interpolated the crossing time t∗ and added only the portion of the interval that was above (for O_2_ER) or below (for DO_2_i) τ.

We quantified the area above O_2_ER thresholds as the time integral of the exceedance (x-τ) + using the trapezoidal rule on each interval: area i = Δt. [(x_i_ - τ)+ + (x_i_ + 1 - τ)_+_]/2, with the same linear interpolation at crossings to truncate triangles exactly at τ. Time is reported in minutes; area, in (unit × minutes).
